# White Blood Cell and C-Reactive Protein Levels Are Similar in Obese Hispanic White Women Reporting Adherence to a Healthy Plant, Unhealthy Plant, or Animal-Based Diet, unlike in Obese Non-Hispanic White Women

**DOI:** 10.3390/nu16040556

**Published:** 2024-02-17

**Authors:** Anna Bruins, Jacob Keeley, Virginia Uhley, Kimberly Anyadike, Kyeorda Kemp

**Affiliations:** 1 Trinity Health Grand Rapids Family Medicine Residency, 200 Jefferson Ave SE, Grand Rapids, MI 49503, USA; anna.e.bruins@trinity-health.org; 2 Department of Research, Oakland University William Beaumont School of Medicine, 586 Pioneer Dr, Rochester, MI 48309, USA; jkeeley@oakland.edu; 3 Department of Foundational Medical Studies, Oakland University William Beaumont School of Medicine, 586 Pioneer Dr, Rochester, MI 48309, USA; uhley@oakland.edu; 4 Department of Family Medicine and Community Health, Oakland University William Beaumont School of Medicine, Rochester, MI 48309, USA; 5 Oakland University William Beaumont School of Medicine, 586 Pioneer Dr, Rochester, MI 48309, USA; kanyadike@oakland.edu

**Keywords:** dietary patterns, obesity, CRP, WBC, Hispanic

## Abstract

While modifying dietary patterns can reduce the effects of inflammation in obesity, less is known about the impact of dietary patterns on inflammation levels in women of different ethnicities. This study investigated the link between dietary patterns and mediators associated with inflammation, such as C-reactive protein (CRP) and white blood cells (WBCs), among obese Hispanic and Non-Hispanic White women. CRP and WBC counts were extracted from the National Health and Nutrition Examination Survey conducted between 2003 and 2010. Based on their recorded responses to two 24 h recall interviews, individuals were grouped into one of three dietary patterns: healthy plant-based, less healthy plant-based, or animal-based. Comparisons were run between obese Hispanic and Non-Hispanic women assigned to the same dietary pattern groups and between dietary pattern groups within ethnic groups. CRP and WBCs increased in obese Non-Hispanics as dietary patterns moved from healthy plant-based to animal-based (*p*CRP = 0.002 and *p*WBC = 0.017). Regardless of the dietary pattern, CRP and WBC expression were similar in Hispanic women. In addition, WBCs were higher in Hispanics compared to Non-Hispanics when both populations adhered to healthy plant and less healthy plant dietary patterns. The results indicate that dietary patterns may influence Hispanics’ inflammation differently than Non-Hispanics.

## 1. Introduction

Obesity, defined as a body mass index (BMI) ≥ 30, is a public health crisis. The incidence of obesity has increased worldwide over the past 20 years, and it is associated with higher incidences of chronic disease risks. In the U.S., an estimated 42.5% of the adult population was determined to meet the BMI criteria for obesity in 2017–2018, compared with 30.5% in 1999–2000. The rates of obesity are higher for underrepresented minority women [1,2,3,4]. Indeed, the National Health and Nutrition Examination Survey 2017–March 2020 shows 45.7% of women who are Hispanic, an ethnic category that encompasses individuals with origins in Spain or other Spanish-speaking countries, are reported to be obese, compared to 39.6% of Non-Hispanic White women [5].

The disease process that links the development of obesity-related comorbidities is described by the underlying inflammatory processes associated with excess adipose tissue. Adipose tissue has functions beyond serving as the main site of excess energy storage. It also functions as an endocrine organ, releasing adipose-derived secreted factors, or adipokines, which influence inflammatory activity [6]. Excess macronutrients and the accumulation of fatty acids in obesity lead to adipose tissue triggering the production of C-reactive protein (CRP) and proinflammatory cytokines. These inflammatory responses subsequently contribute to elevated white blood cell (WBC) counts, altogether fostering an inflammatory environment [7]. An inflammatory environment is linked to various metabolic diseases and puts individuals at higher risk of health conditions, ranging from arthritis and sleep apnea to diabetes and stroke. Ultimately, the aggregate national cost of obesity in the U.S. is estimated to be USD 260.6 billion, with adults with obesity in the U.S. experiencing higher annual medical care costs—USD 2505 more or 100% of the cost for non-obese individuals [8]. This further necessitates identifying interventions that can reduce the rate of and impact of obesity on health risks. Moreover, because the rates of obesity continue to rise, mechanisms for reducing obesity-associated disorders must be investigated in an effort to decrease the burden of obesity in America with targeted therapies, keeping in mind the population groups who are most impacted.

The causes of obesity are multifactorial and complex, influenced by a combination of behavioral patterns, cultural practices and beliefs, hormonal regulation, genetic factors, socioeconomic factors, and structural discrimination [4,9,10,11]. However, extensive research on nutrition indicates that dietary patterns play a role in obesity and that diet impacts chronic disease health risks. Dietary patterns that emphasize nutrient-dense foods with a focus on plant-based foods have been reported to reduce the risks of developing cardiovascular disease, type 2 diabetes, cancer, and all-cause mortality, all of which are associated with elevated inflammatory markers [12,13,14]. In addition, plant-based dietary patterns have been shown to be associated with reduced levels of oxidative stress and inflammatory markers [15]. Moreover, proinflammatory nutritional components and anti-inflammatory nutrition components have been shown to impact chronic disease risks [16]. Fortunately, dietary patterns of intake can be modified to reduce the impact of inflammation caused by obesity [17].

Exploring how dietary patterns impact markers of inflammation in Hispanic populations is of particular interest, as obesity-related comorbidities occur more frequently in this population compared to White and Asian populations [1,3,18]. Moreover, the Hispanic population is the second-largest ethnic group in the United States, with a population of 62.1 million people in 2020 [19], and the growth is ongoing. Between 2010 and 2019, the Hispanic population contributed more significantly to the overall growth of the nation than any other ethnic group [19]. Therefore, as the U.S. population continues to evolve, the need to address escalating health disparities among this population is pivotal.

Plant-based diets have been recognized as a successful approach to mitigating obesity and its associated health risks [20,21]. However, recent studies focusing on Hispanic individuals suggest that following a plant-based dietary pattern may not have the same impact on inflammation, weight loss, and disease risk as observed in broader population studies. In a study conducted by Singh et al. that investigated plant-based diets in a limited group of Hispanic Seventh-Day Adventists, non-vegetarians were observed to have greater adiposity than vegetarians. Yet, IL-6 and CRP levels were similar regardless of diet [22]. Zuercher et al. (2023) found that adherence to a more inflammatory diet (i.e., higher intake of processed foods, sweets, and processed meats) elevated the risk of stroke and cardiovascular issues in overweight Hispanic women [23]. Interestingly, this association was not observed in either obese or normal-weight Hispanic women. Additionally, the relationship between dietary patterns and inflammatory mediators was found to be less pronounced in postmenopausal women of color compared to White women [24]. Finally, Arias-Gastélum et al. found that adherence to a plant-and-fish-based dietary pattern was associated with an increase in fasting blood glucose in obese and overweight Hispanic women at risk of or already having type 2 diabetes [25]. However, this finding is in conflict with earlier studies demonstrating that implementing a plant-based dietary intervention reduces fasting blood glucose [26], and prior work has shown that adhering to a healthy plant-based dietary pattern reduces diabetes risk in Puerto Ricans [27].

These published reports indicate that the benefits of plant-based diets must be further explored in underrepresented minority women. This study explores how WBCs and CRP are expressed in obese Non-Hispanic White (Non-Hispanic) and Hispanic White (Hispanic) women reporting majority adherence to healthy plant-based, less healthy plant-based, and animal-based dietary patterns. We find that, as previously published, CRP and WBC levels are higher in Non-Hispanics reporting an animal-based dietary pattern when compared to those reporting a healthy plant-based dietary pattern; however, CRP and WBC levels are similar in Hispanics, regardless of reported dietary pattern.

## 2. Materials and Methods

### 2.1. Sample

Data were collected from the nationally representative cross-sectional surveys of the U.S. non-institutionalized civilian population conducted by the U.S. National Center for Health and Statistics (National Health and Nutrition Examination Survey (NHANES), 2003/2004 to 2009/2010). NHANES has collected information regarding demographics, lifestyle, nutritional habits, and laboratory values from nationally representative samples in the U.S. in two-year cycles since 1960. NHANES uses robust and multistage probability cross-sectional sampling to ensure that participant selection occurs from various geographical locations across the U.S. and that various racial/ethnic groups are included. An in-home interview is conducted with NHANES participants regarding behavior, diet, socioeconomic factors, etc. Individuals are then invited to their mobile examination clinic, where laboratory tests are run. Please see the NHANES website for more information: https://www.cdc.gov/nchs/nhanes/index.htm?CDC_AA_refVal=https%3A%2F%2Fwww.cdc.gov%2Fnchs%2Fnhanes.htm (accessed on 23 November 2021). Subjects were included in the analysis if they had two separate interviews pertaining to their daily food intake and were identified as White women over 18 years of age. 

### 2.2. Data Extracted

Variables specific to the study were collected and merged from multiple different NHANES datasets within a given two-year data cycle. The datasets included the Demographics files, Body Measures files, Dietary Interview files, and Laboratory files. Four cycles of two-year data, 2003–2004, 2005–2006, 2007–2008, and 2009–2010, were then combined in order to increase the amount of data for analysis.

#### 2.2.1. BMI and Waist Circumference

BMI and waist circumference were extracted from the Body Measures file. Individuals were then separated into obese (BMI ≥ 30) and non-obese categories (BMI < 30), and those who were obese were included in the study. These measures were identified due to the extensive use and validation of these measures to define obesity, including in Hispanic populations [28,29,30]. Moreover, BMI and waist circumference have a strong correlation with other obesity measures, such as waist-to-height ratio, for Hispanic women [28].

#### 2.2.2. Dietary Pattern

**Figure 1:**
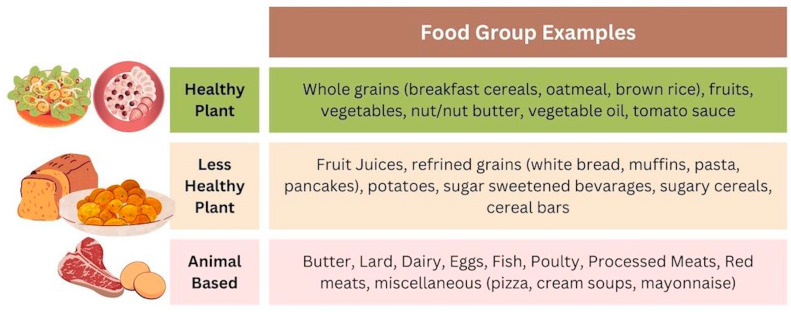
Dietary pattern groups. Food assignments were adapted from Satija et al., 2017 [30].

The data collection protocols for NHANES have been described elsewhere [29]. The subjects in this study were categorized as following one of three dietary patterns, i.e., healthy plant food groups, less healthy plant food groups, and animal food groups, according to their food interviews. In order to create the three dietary pattern groupings, food intake identified in the dietary 24 h recall listed in the interview was first classified into 1 of 18 groupings as in Satija et al. [30]. Of the 18, 6 (animal fat, dairy, egg, fish/seafood, meat, and miscellaneous animal-based) were labeled as an animal food dietary pattern, 5 (fruit juices, refined grains, potatoes, sugar-sweetened beverages, and sweet/desserts) were labeled as a less healthy plant dietary pattern, and 7 (whole grains, fruits, vegetables, nuts, legumes, vegetable oils, and tea/coffee) were labeled as a healthy plant food dietary pattern (Figure 1). Lastly, the number of times each of the three groups appeared per subject was calculated. Subjects having the majority of their food interview represented as healthy plant food groups, less healthy food groups, or animal food groups were categorized as such. Where there was no majority to be found, subjects were categorized as less healthy plant groups.

#### 2.2.3. Laboratory Values

Data related to total WBCs and CRP were isolated from the Laboratory files. These values were chosen because they have been previously established as reliable markers for inflammation, and dietary pattern has a strong relationship with these markers [16,31,32].

#### 2.2.4. Demographics

Information regarding ethnicity and age was collected from the Demographics files. Those who were identified in the NHANES database as Mexican American or other Hispanic were combined into Hispanic.

### 2.3. Statistical Analysis

We were interested in exploring the impact of dietary patterns and ethnicity on WBCs and CRP. To achieve this, we had to compare across groups. Therefore, lab values were first summarized overall and then stratified by ethnicity and diet. To determine whether there was a statistically significant difference between the mean lab values between both ethnicities and dietary patterns, multiple two-way ANOVAs with interactions were performed for WBCs and CRP, respectively. When there was evidence of a statistically significant interaction between ethnicity and dietary pattern, post hoc testing was performed to identify where the statistically significant differences were between groups. Multiple testing was conducted using a Tukey–Kramer adjustment when necessary.

All analyses took into account the complex sampling design utilized by NHANES, using the appropriate weights for subjects who participated in dietary interviews. For details on the sampling procedures used, visit the NHANES website at http://www.cdc.gov/nchs/nhanes.htm (accessed on 23 November 2021) Tests were two-sided using an alpha of 0.05. All analyses were performed using SAS v9.4, specifically using the SURVEY procedures.

## 3. Results

### 3.1. Higher WBC Count in Obese Hispanic Women despite Younger Age and Higher Numbers of Individuals Reporting a Healthy Plant Dietary Pattern

The demographic data are reported in Table 1 regarding WBCs, CRP, BMI, age, waist circumference, and dietary pattern for all individuals, Hispanic women, and Non-Hispanic women. Overall, the age, BMI, and waist circumference differed significantly between the two groups, with Hispanic women trending younger (41.49 vs. 50.20) and having a lower BMI (35.68 vs. 36.34) and waist circumference (112.02 vs. 108.58) (*p* < 0.001, 0.015, and <0.001, respectively). Hispanic women had higher levels of WBCs compared to Non-Hispanic women (*p* = 0.002); however, CRP levels were similar (*p* = 0.561). Moreover, the reported dietary patterns differed significantly (*p* = 0.004), with similar numbers of individuals reporting a less healthy plant-based dietary pattern; however, 10% of Hispanic women reported a healthy plant-based dietary pattern vs. 6.9% of Non-Hispanic women.

### 3.2. WBC Count Is Higher in Obese Hispanic Women Reporting a Healthy and Less Healthy Plant Dietary Pattern Compared to Non-Hispanic Women Reporting the Same Dietary Pattern

**Figure 2:**
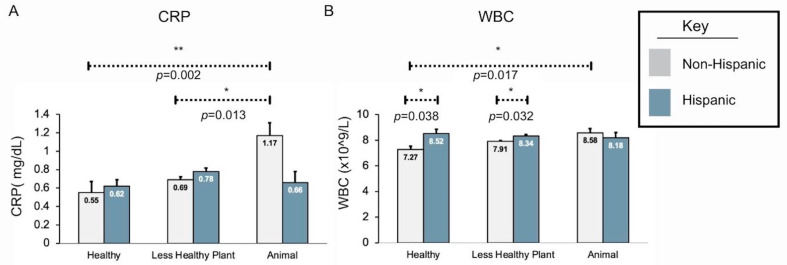
Diet influences inflammatory marker expression in obese Non-Hispanic women differently than in obese Hispanic White women. (**A**) CRP is significantly elevated in Non-Hispanic women (gray bars) when comparing healthy plant-based and animal-based dietary patterns or less healthy plant-based and animal-based. (**B**) WBC is significantly elevated in Non-Hispanic women (gray bars) when comparing healthy plant-based and animal-based dietary patterns. In obese Hispanic women (blue bars), the dietary pattern does not significantly alter CRP (**A**) and WBC (**B**) expression. Solid lines indicate a 2-way ANOVA between six independent dietary pattern and ethnicity groups. Dashed lines indicate an independent sample *t*-test between dietary pattern types within the same ethnic group or an independent sample t-test between ethnic groups within the same diet type. * = *p* < 0.5, ** = *p* < 0.01.

The two-way ANOVA with ethnicity and dietary pattern groups using CRP as the outcome suggested evidence of an interaction between the two variables (*p*-value = 0.008). Pairwise comparisons were examined during post hoc testing. Within specific dietary pattern groups, there did not appear to be any statistically significant differences between the Hispanic and Non-Hispanic groups based on CRP levels (Figure 2 and Tables S3 and S4).

When analyzing both ethnicity and dietary patterns in a two-way ANOVA using WBC count as the outcome, there was statistically significant evidence of an interaction between the two variables (*p*-value = 0.025; Figure 2). Post hoc testing suggested that Hispanic women reporting a healthy plant-based dietary pattern had an elevated WBC count of 1.25 (adjusted *p*-value = 0.038) compared to Non-Hispanic women. The difference in WBC count was 0.43 when comparing Hispanic and Non-Hispanic women reporting a less healthy plant-based dietary pattern (adjusted *p*-value = 0.032). There was no statistically significant difference in WBC count based on ethnicity for those who reported following an animal-based dietary pattern (adjusted *p*-value = 0.941).

### 3.3. WBCs and CRP Are Elevated in Obese Non-Hispanic Women Reporting an Animal-Based Dietary Pattern Compared to Those Reporting a Healthy Plant Dietary Patter, Unlike Hispanic Women, Where There Is No Difference

When analyzing CRP within the Non-Hispanic comparisons, there were differences between the healthy plant-based and animal-based dietary pattern groups, as well as the less healthy plant-based and animal-based dietary pattern groups. The healthy plant-based dietary pattern group had a decrease of 0.62 (adjusted *p*-value = 0.002) in CRP when compared to the animal dietary pattern group, whereas the less healthy plant-based dietary pattern groups had a decrease of 0.48 (adjusted *p*-value = 0.013) from the animal-based dietary pattern group. The comparison between healthy and less healthy dietary patterns within the Non-Hispanic group did not suggest a significant difference (adjusted *p*-value = 0.858). Last, again, there were no statistically significant differences between different dietary patterns when considering the Hispanic group (Figure 2 and Tables S3 and S4).

Within the Non-Hispanic sample, the WBC count is lower for those reporting a healthy plant-based dietary pattern compared to an animal-based dietary pattern (decrease of 1.31, adjusted *p*-value = 0.0174). There was no statistically significant difference between animal-based and less healthy plant-based dietary patterns or less healthy and healthy plant-based dietary patterns (adjusted *p*-values = 0.384 and 0.151, respectively). Interestingly, there were no statistically significant differences between dietary pattern groups among Hispanic women (Figure 2 and Tables S1 and S2).

## 4. Discussion

This study illuminates crucial connections between ethnicity, dietary patterns, and key inflammatory biomarkers, i.e., WBCs and CRP, in the context of obesity. While previous research has shown that adopting a plant-based diet can mitigate the effects of obesity, inflammation, and related morbidities [15], many of these studies did not specifically focus on minority populations. Recent nutrition studies exploring the impact of dietary patterns on inflammation and disease among minority groups suggest that adherence to a plant-based diet may yield different outcomes regarding inflammation, weight loss, and disease compared to broader multiethnic population studies [33]. Indeed, our analysis of obese Hispanic women revealed a distinct pattern. Unlike obese Non-Hispanic women, where reported adherence to a healthy plant-based dietary pattern led to lower CRP levels, the CRP levels in obese Hispanic women remained similar regardless of the reported dietary pattern. Interestingly, it was also observed that while the WBC count increased in Non-Hispanic women when comparing a healthy plant-based pattern to an animal-based one, the WBC count trended in the downward direction in Hispanic women. This study contributes valuable insights to the expanding body of literature in this field.

Obesity promotes a proinflammatory metabolic state, thereby increasing health risks. Adipose tissue can produce many proinflammatory adipokines and cytokines that foster oxidative stress, adversely affecting health status [34,35]. Elevated levels of proinflammatory markers, such as CRP and WBC, have been observed in obese individuals and correlate with the development of a number of disease states [6,7,36]. However, it is important to note that obesity is not the only factor that can increase inflammatory markers. Indeed, factors such as sleep, daily stressors, discrimination, socioeconomic factors, and access to health professionals can all impact inflammation levels [37,38,39]. In addition, there is evidence that there are differences in metabolism amongst ethnic groups that can impact obesity, inflammation, and the development of disease [40].

Studies indicate that underrepresented minorities have higher levels of proinflammatory markers compared to their counterparts [41,42]. African-Americans are consistently found to have higher levels of inflammatory markers such as CRP, while this differs for Hispanic individuals. Some studies indicate that inflammatory markers are higher than in Hispanic Whites, while others indicate that the rates are similar [43,44]. Moreover, a study exploring inflammation in children found elevated CRP in Hispanic and African-American children compared to Non-Hispanic White children, even when accounting for various factors, including demographics, illness, and birth conditions [41]. This was even more pronounced for those with parents who were foreign-born. Nativity, acculturation, and immigration history appear to impact inflammation levels in adults as well [39,45,46]. Inflammation levels were lower for newly immigrated individuals with ties across the U.S. border, while U.S.-born individuals without cross-border ties had higher levels of inflammation [45]. Martin et al. found that second- and third-generation Mexican Americans had lower levels of inflammation compared to first-generation individuals. Moreover, inflammation was lower in first-generation individuals residing in the U.S. for 15 years compared to those residing there for more than 15 years. It may be that social connections or cultural practices may be protective. It is also possible that the adoption of a less healthy diet may play a role, as individuals who immigrated to the U.S. in early or middle childhood had a less healthy diet than those who arrived during adulthood [47]. Hence, those with reduced cultural ties through the process of acculturation adopt a less healthy diet [48,49,50].

While acculturation negatively impacts diet and inflammation, stressors such as adverse childhood events, finances, discrimination, adverse neighborhood events, and employment also play a role. A recent study exploring inflammation in White, Asian, African-American, and Hispanic individuals with the same occupation (i.e., nursing) in the same region of the country found that inflammation levels were similar [51]. This may be because there were similar stressors at play within this population. Cuevas et al. determined that Hispanic, African-American, and White individuals had similar levels of obesity if socioeconomic factors, diet, and physical activity were the same [52]. However, exposure to three stressors, regardless of the type, increased the prevalence of obesity in Hispanics by 14% compared to White individuals. In this study, we found that even though obese Hispanic women had a lower BMI (35.7 vs. 36.3, *p* = 0.015), younger age (41.5 vs. 50.2, *p* < 0.001), and smaller waist circumference (112.0 vs. 108.6, *p* < 0.001) than obese Non-Hispanic women, they had higher levels of WBCs compared to the obese Non-Hispanic women and similar levels of CRP. This finding is interesting, as BMI, age, and waist circumference are inversely associated with inflammation [53,54,55]. This finding suggests that factors beyond obesity may be influencing inflammatory markers [7,56].

Even though a higher percentage of obese Hispanic women reported consuming a healthy plant-based dietary pattern, overall, they had higher WBCs compared to obese Non-Hispanic women. This indicates that a healthy plant-based dietary pattern did not have the same impact on inflammatory markers. In addition, the type of dietary pattern (healthy plant-based, less healthy plant-based, or animal-based) did not have an impact on CRP levels in obese Hispanic women in this study. These findings align with a study by Edgar et al. conducted on Mexicans living in an urban area. Their findings revealed that following a dietary pattern closely resembling a healthy plant-based diet was associated with a reduced risk of cardiovascular disease. In contrast, adherence to a meat/fish dietary pattern did not show any impact on cardiovascular disease risk [57].

Sources and types of fat and protein in food vary in individuals by race and ethnicity. A study on Puerto Ricans determined that the food source of fat and protein in the diet was an important predictor of cardiovascular risk [58]. A higher intake of fish, seafood, and unprocessed meat was associated with a reduced waist circumference and lower triglyceride levels. Concurrently, serum trans-fatty acids demonstrated a positive association with CRP in both Hispanic White and Non-Hispanic White individuals [59]. It is possible that the types of fat and protein varied in the animal-based diets within the populations in this study. For example, Hispanics, as a group, consume more overall protein, with a notable emphasis on unprocessed red meat and eggs, compared to their Non-Hispanic White counterparts [29].

While many studies have reported the positive impact of adopting healthy plant-based dietary patterns in reducing inflammation and lowering the risk of disease in Hispanics, conflicting findings exist. Complicating matters further is the ambiguity in the descriptions of plant-based dietary patterns used in interventions, making it challenging to compare and reproduce research studies [60]. Furthermore, our analysis does not compare individuals against an index or strict adherence as in other studies [29,61,62,63]. While this approach might obscure differences within dietary patterns, it may offer a more accurate depiction of real-world behaviors, as a growing number of individuals have adopted a more flexible approach to diet [61,64]. In addition, other factors may play a role, such as variable eating timing patterns, which have also been reported to be associated with increases in CRP levels [65] or excessive caloric intake. Recently, Arias-Gastelum et al. reported that a higher intake of plant foods and fish resulted in an increase in fasting blood glucose levels in overweight/obese Hispanic women with established risk factors for type 2 diabetes who adhered to healthy dietary patterns [25]. They suggested that it may have been that the total caloric intake was excessive in comparison to the other dietary patterns and/or that the women with altered glucose levels might have been attempting to adhere to a dietary pattern with healthier traits. This is also a possibility for our study.

## 5. Limitations

We used BMI to classify individuals as obese in this study. Although there remains controversy regarding the accuracy of BMI for determining obesity and the associated health risks, BMI is considered a commonly used tool for measuring obesity and the propensity to develop chronic disease [66]. The main controversy with this measure is that BMI does not distinguish between muscle and fat accumulation [67,68]. BMI also does not distinguish body fat distribution; alternatively, the use of waist circumference (WC) measures has been shown to be an indicator of health risks associated with obesity [69,70]. It is possible that the use of the BMI metric led to individuals being categorized as obese who were not. However, the mean waist circumferences of both Hispanic and Non-Hispanic individuals in this study are well above the cutoffs previously established for obesity and obesity-related disease risks, including for Hispanic individuals [71,72,73]. In addition, waist circumference was previously shown to have a very strong correlation with BMI (0.87) in Hispanic women [28].

The use of dietary recall has the limitation of recall bias, which can lead to an under- or overestimation of food and nutrient intake, and some variables, such as food codes and descriptions, changed from year to year within the database. We did not analyze the quantity of food and caloric intake, and it is possible that this may have impacted our data.

Hispanics are not monolithic. Indeed, the term Hispanic refers to more than twenty countries, and within those countries exist a wealth of rich cultures and associated practices. Differences exist between Mexicans, individuals from the various diverse Caribbean Nations, and Latin Americans with regard to food makeup, which may impact the analysis. NHANES reports the demographic category as “Mexican and other”, which we combined. Because Mexicans make up the majority of the population, these results may mask the association between dietary patterns and inflammation within this varied ethnic group. For example, a 2017 study found that Puerto Ricans consumed diets low in fiber and rich in fruit, fruit juice, and beans, while Mexican Americans consumed diets rich in vegetables, beans, and total grains [74]. Therefore, these findings and others may be impacted by the makeup of the groups categorized as Hispanic. Dietary interventions designed for Hispanic populations may need to be tailored to be culturally specific for unique makeups.

Lastly, we do not account for lifestyle factors, medications, comorbidities, or hormonal changes influenced by menopause, childbirth/breastfeeding, or birth control. The focus of this study was to explore whether there were differences between inflammatory mediators in individuals reporting different dietary patterns. Future studies will further explore the impact of dietary patterns on inflammation when accounting for these factors. While ethnic disparities in immune response related to obesity were discerned, caution is warranted, as the strength of these differences may require further exploration.

## 6. Conclusions

This study finds that the association between healthy plant-, less healthy plant-, or animal-based dietary patterns and inflammatory markers differs based on ethnicity in obese women. These nuanced findings advocate for tailored interventions that acknowledge both ethnic and dietary influences on obesity-associated health risks. It is crucial to interpret these associations prudently, recognizing the need for additional research to establish causation and refine targeted interventions for effective obesity management.

## Figures and Tables

**Table 1:** Demographic data for the population overall and by ethnicity.

	Overall	Non-Hispanic	Hispanic ^1^	*p*-Value
Age—Mean (SE) N (Weighted N)	48.69 (0.53) 2651 (31,233,652)	50.20 (0.66) 1569 (25,820,642)	41.49 (0.61) 1082 (5,413,010)	<0.001 ^2^
BMI—Mean (SE) N (Weighted N)	36.23 (0.20) 2651 (31,233,652)	36.34 (0.23) 1569 (25,820,642)	35.68 (0.18) 1082 (5,413,010)	0.015 ^2^
Waist Circumference (cm)—Mean (SE) N (Weighted N)	111.42 (0.48) 2750 (30,298,973)	112.02 (0.53) 1513 (24,945,847)	108.58 (0.48) 1057 (5,293,126)	<0.001 ^2^
WBC Count—Mean (SE) N (Weighted N)	8.01 (0.07) 2572 (30,279,467)	7.93 (0.08) 1515 (24,996,737)	8.35 (0.10) 1057 (5,282,730)	0.002 ^2^
CRP—Mean (SE) N (Weighted N)	0.74 (0.02) 2550 (29,942,864)	0.73 (0.03) 1497 (24,675,108)	0.76 (0.04) 1053 (5,267,756)	0.561 ^2^
Reported Dietary Pattern				0.004 ^3^
Healthy Plant	223 (2,315,262) 7.41%	117 (1,774,678) 6.87%	106 (540,585) 9.99%	
Less Healthy Plant	2224 (26,031,238) 83.34%	1301 (21,455,217) 83.09%	923 (4,576,020) 84.54%	
Animal	204 (2,887,152) 9.24%	151 (2,590,747) 10.03%	53 (296,405) 5.48%	

All statistical analyses were performed between Hispanic and Non-Hispanic populations for the variables listed. The CRP unit is mg/dL, and the WBC unit is 10^9^/L. ^1^ The Mexican and other Hispanic categories were combined. Mexican *n* = 809, Weighted *n* = 3,812,780 (70.44%); other Hispanic *n* = 273, Weighted *n* = 1,600,230 (29.56%). ^2^ Two-sample *t*-test. ^3^ Rao Scott Chi-Sq Test.

## Data Availability

Data are available in a publicly accessible repository. The data presented in this study are openly available through the Centers for Disease Control and Prevention (CDC). National Center for Health Statistics (NCHS). National Health and Nutrition Examination Survey Data. Hyattsville, MD: U.S. Department of Health and Human Services, Centers for Disease Control and Prevention, (2003–2010) [https://wwwn.cdc.gov/nchs/nhanes/Default.aspx, (accessed on 23 November 2021).

## Supplementary Materials

The following supporting information can be downloaded at https://www.mdpi.com/article/10.3390/nu16040556/s1: Tables S1 and S3 contain the means and SE for WBCs and CRP shown in Figure 2. Tables S2 and S4 contain the results of all statistical analyses performed.

## Abbreviations

National Health and Nutrition Examination Survey (NHANES), body mass index (BMI), C-reactive protein (CRP), and white blood cell (WBC) counts.
