# Supplementary material for: White Blood Cell and C-Reactive Protein Levels Are Similar in Obese Hispanic White Women Reporting Adherence to a Healthy Plant, Unhealthy Plant, or Animal-Based Diet, unlike in Obese Non-Hispanic White Women
Source: Nutrients. 2024 Feb 17;16(4):556. doi: 10.3390/nu16040556 (PMC10891662; doi:10.3390/nu16040556)
Supplement: Supplementary file 1 [file nutrients-16-00556-s001.zip › nutrients-2857427-supplementary.pdf]

Table S1: WBC Table

	Non-Hispanic<br>N (Weighted N)	Hispanic<br>N (Weighted N)
Healthy Plant – Mean (SE)	7.27 (0.26)	8.52 (0.32)
Less Healthy Plant – Mean (SE)	7.91 (0.07)	8.34 (0.11)
Animal – Mean (SE)	8.58 (0.34)	8.18 (0.41)

Table S2: WBC Comparisons

	Within	Difference	P-Value
Healthy Plant vs. Less Healthy Plant	Hispanic	0.17 (0.33)	0.9949
Healthy Plant vs. Animal	Hispanic	0.34 (0.54)	0.9876
Less Healthy Plant vs. Animal	Hispanic	0.17 (0.43)	0.9987
Healthy Plant vs. Less Healthy Plant	Non-Hispanic	-0.64 (0.26)	0.1507
Healthy Plant vs. Animal	Non-Hispanic	-1.31 (0.39)	0.0174
Less Healthy Plant vs. Animal	Non-Hispanic	-0.67 (0.34)	0.3838
Hispanic vs. Non-Hispanic	Healthy Plant	1.25 (0.41)	0.0381
Hispanic vs. Non-Hispanic	Less Healthy Plant	0.43 (0.14)	0.0317
Hispanic vs. Non-Hispanic	Animal	-0.40 (0.44)	0.9411

Table S3: CRP Table

	Non-Hispanic	Hispanic
Healthy Plant – Mean (SE)	0.55 (0.12)	0.62 (0.07)
Less Healthy Plant – Mean (SE)	0.69 (0.03)	0.78 (0.04)
Animal – Mean (SE)	1.17 (0.14)	0.66 (0.12)

Table S4: CRP – Comparisons

	Within	Difference	P-Value
Healthy Plant vs. Less Healthy Plant	Hispanic	-0.16 (0.08)	0.2737
Healthy Plant vs. Animal	Hispanic	-0.04 (0.14)	0.9997
Less Healthy Plant vs. Animal	Hispanic	0.13 (0.13)	0.9221
Healthy Plant vs. Less Healthy Plant	Non-Hispanic	-0.14 (0.12)	0.8579
Healthy Plant vs. Animal	Non-Hispanic	-0.62 (0.15)	0.0020
Less Healthy Plant vs. Animal	Non-Hispanic	-0.48 (0.14)	0.0126
Hispanic vs. Non-Hispanic	Healthy Plant	0.07 (0.14)	0.9956
Hispanic vs. Non-Hispanic	Less Healthy Plant	0.09 (0.05)	0.5428
Hispanic vs. Non-Hispanic	Animal	-0.51 (0.19)	0.0855
